# Sequence-structure-function characterization of the emerging tetracycline destructase family of antibiotic resistance enzymes

**DOI:** 10.1038/s42003-024-06023-w

**Published:** 2024-03-16

**Authors:** Kevin S. Blake, Hirdesh Kumar, Anisha Loganathan, Emily E. Williford, Luke Diorio-Toth, Yao-Peng Xue, Wai Kwan Tang, Tayte P. Campbell, David D. Chong, Steven Angtuaco, Timothy A. Wencewicz, Niraj H. Tolia, Gautam Dantas

**Affiliations:** 1 The Edison Family Center for Genome Sciences and Systems Biology, Washington University School of Medicine, St. Louis, MO USA; 2 Host-Pathogen Interactions and Structural Vaccinology section (HPISV), National Institute of Allergy and Infectious Diseases (NIAID), National Institutes of Health (NIH), Bethesda, MD USA; 3 Department of Chemistry, Washington University in St. Louis, St. Louis, MO USA; 4 Department of Pathology and Immunology, Division of Laboratory and Genomic Medicine, Washington University School of Medicine, St. Louis, MO USA; 5 Department of Molecular Microbiology, Washington University School of Medicine, St. Louis, MO USA; 6 Department of Biomedical Engineering, Washington University School of Medicine, St. Louis, MO USA; 7 Department of Pediatrics, Washington University School of Medicine, St. Louis, MO USA

**Keywords:** Bacterial genes, Antibiotics

## Abstract

Tetracycline destructases (TDases) are flavin monooxygenases which can confer resistance to all generations of tetracycline antibiotics. The recent increase in the number and diversity of reported TDase sequences enables a deep investigation of the TDase sequence-structure-function landscape. Here, we evaluate the sequence determinants of TDase function through two complementary approaches: (1) constructing profile hidden Markov models to predict new TDases, and (2) using multiple sequence alignments to identify conserved positions important to protein function. Using the HMM-based approach we screened 50 high-scoring candidate sequences in *Escherichia coli*, leading to the discovery of 13 new TDases. The X-ray crystal structures of two new enzymes from *Legionella* species were determined, and the ability of anhydrotetracycline to inhibit their tetracycline-inactivating activity was confirmed. Using the MSA-based approach we identified 31 amino acid positions 100% conserved across all known TDase sequences. The roles of these positions were analyzed by alanine-scanning mutagenesis in two TDases, to study the impact on cell and in vitro activity, structure, and stability. These results expand the diversity of TDase sequences and provide valuable insights into the roles of important residues in TDases, and flavin monooxygenases more broadly.

## Abstract (web summary)

Deep investigation of the sequence-structure-function landscape of the tetracycline destructase family of antibiotic resistance enzymes identifies new enzymes, and amino acid spositions essential to activity.

## Introduction

Tetracycline antibiotics have been extensively used in the clinic and agriculture since their discovery over 70 years ago^1–3^. Predictably, this selective pressure has resulted in the expansion of the tetracycline resistome^4–7^. Tetracycline resistance in clinical samples has most frequently occurred by efflux pumps and ribosomal protection proteins^2,6,8,9^. Resistance by these mechanisms was countered by the development of third-generation tetracyclines (i.e., glycylcyclines), including tigecycline, omadacycline, eravacycline, and sarecycline—the latter three approved by the US FDA in 2018^10–14^. Tigecycline is used as a “last resort” treatment reserved for infections by multidrug-resistant pathogens such as *Acinetobacter baumannii*^9,15^. However, the continued utility of all generations of tetracycline antibiotics is threatened by the emergence of a third mechanism of resistance: tetracycline-inactivating enzymes^14^.

Tetracycline destructases (TDases) are class A flavin monooxygenases (FMO) that covalently modify and inactivate tetracycline antibiotics^7^ (Supplementary Fig. 1). These enzymes are categorized into two types based on habitat, sequence similarity, and activity profiles^16^. Type 1 TDases include the prototypical Tet(X), and are predominantly found in human gut microbiomes and pathogens, including *Acinetobacter baumannii*, *Pseudomonas aeruginosa*, *Enterobacter cloacae*, *Klebsiella pneumoniae*, and *Escherichia coli*^17–21^. Type 2 TDases have thus far been predominantly identified through soil functional metagenomics with one sequence found in the soil-derived human pathogen *Legionella longbeachae*^7,22,23^. These two types differ in their resistance profiles, with type 1 enzymes conferring resistance to all tetracycline antibiotics including third-generation drugs while type 2 enzymes are generally limited to only first- and second-generation tetracyclines^21^. Further, the two types represent two distinct sequence clades with low (~20%) amino acid identity to each other^7,21^. A far greater number of type 1 TDases have been sequenced and characterized to date, due largely to tigecycline screening of functional metagenomic libraries and isolates^18–21^. In contrast, the type 2 sequences, although fewer in number, show far greater intra-clade sequence diversity^21^. Despite these differences in habitat, resistance activity profiles, and sequence identity, the two TDase types have a similar overall tertiary structure in line with the class A family of FMOs^24^. Each possesses a FAD-binding Rossmann-type fold domain (FBD), a substrate- (tetracycline) binding domain (SBD), and a C-terminal α-helix that structurally bridges the two domains^23^.

Tetracycline resistance determinants, generally, are designated “tet” followed by a letter or number^25^. All type 1 TDases discovered thus far are variants (79-98% amino acid identity) or sub-variants (98-99.9% amino acid identity) of the Tet(X) gene. Type 1 gene variants are indicated by a number after the “X”, and sub-variants by a period followed by another number (e.g., Tet(X2) and Tet(X2.2)). There are 10 type 2 TDase determinants discovered thus far, and these are numbered Tet(47) to Tet(56). Type 2 gene variants are designated by a hyphen followed by a number, and sub-variants by a period followed by another number (e.g., Tet(50-2) and Tet(50-2.2)).

While the first tetracycline-resistant bacterium was isolated in 1953^6^, the first TDase was not discovered until 1991^26^. For nearly 30 years, Tet(X) and Tet(X2)^8,27^—which differ by just one amino acid substitution—were the only reported TDase sequences. Therefore, it had been presumed that these enzymes were relatively uncommon and unlikely to impact the clinical effectiveness of third-generation tetracyclines^28^. However, that paradigm has been upended in recent years with a flurry of reports describing newly discovered TDase sequences, beginning in 2015 with the discovery of 10 type 2 sequences^7^, and continuing in 2019-2020 with reports totaling over 20 new type 1 TDases^20,21,29–34^. These reports have shown that TDases are globally distributed^7,35^, can be harbored by inducible and mobile plasmids^9,18–20,36^, are carried by MDR human pathogens classified as “urgent threats”^19,35^, and can be colocalized with the carbapenemase *blaNDM-1*^37^ and the colistin resistance gene *mcr-1*^38^. Many of these TDase sequences were discovered by reanalyzing existing sequencing datasets or rescreening previously collected isolates, demonstrating that TDases have been circulating unnoticed for decades. This highlights a fundamental challenge of linking genotype to phenotype despite the rapid expansion of DNA sequence databases, and suggests that within the immense sequence diversity of the FMO family of enzymes (>18,000 sequences in PFAM PF00743) the TDase family may have greater diversity than is currently appreciated^14^. Additionally, reports have shown that single amino acid substitutions in TDases can provide gain-of-function under tigecycline selection^39^. Thus, broad monitoring for new TDase sequences, even variants with only one substitution, is critical for the proactive management of this emerging mechanism of tetracycline resistance.

Additionally, the growing number of known, functionally characterized TDases permits the study of more fundamental aspects of TDase enzymology, including the sequence determinants of their tetracycline-inactivating function. The sequence-structure-function paradigm states that a protein’s primary sequence dictates its three-dimensional structure, which in turn determines its function^40,41^. As residues critical for enzyme function tend to be conserved across protein families, sequence alignments are used to identify these sites^42–44^. According to the neutral theory of molecular evolution, substitution at sites less relevant for function will be selectively neutral (or only slightly deleterious) and because the protein can tolerate changes at these sites, the sites will be variable in sequence alignments. In contrast, because substitution of sites important for function will result in loss of function and natural selection will remove these deleterious mutants, critical residues will be conserved in sequence alignments^45^. Therefore, because functional constraints dictate how conserved a position is, conservation indicates a position is functionally important^46^. Further, previous reports have indicated that these important residues are generally limited in number, as evidenced by structurally similar proteins having low sequence identity and the ability to experimentally mutate many positions along a protein sequence without loss of activity^47^.

Here, we have leveraged the diversity of newly discovered TDase sequences to characterize the TDase family using two complementary sequence-based approaches. First, we used profile hidden Markov models (HMMs) to predict new TDase sequences from public sequence databases. Profile HMMs are probabilistic models that turn an MSA into a position-specific scoring system that quantitatively represents the conservation of each amino acid at each position of the alignment. In contrast to standard BLAST pairwise “top hit” analyses that are biased toward sequences with high overall sequence identity, profile HMMs are well suited to identifying remote homologs with conserved domains but low global pairwise identity^48^. Curated databases of profile HMMs are commonly employed by several sequence annotation programs, such as Pfam/Interpro^49^, AMRFinder^50^, and Resfams^51^. We recombinantly expressed high-scoring HMM hits in *Escherichia coli*, and screened these strains for tetracycline-inactivation activity. Sequences that conferred resistance in these assays were designated bona fide TDases. We determined the X-ray crystal structures of two of these new sequences from *Legionella* species, and confirmed anhydrotetracycline can inhibit these enzymes’ inactivation of tetracycline antibiotics. Second, we evaluated the roles of amino acid sequence positions conserved across all TDases. Using MSAs we identified individual positions that are 100% conserved in 114 functional TDase sequences, and then performed alanine-scanning mutagenesis at these sites in the type 1 TDase Tet(X7) and the type 2 TDase Tet(50), totaling 54 mutant proteins. Screening these mutants on tetracycline antibiotics using whole-cell phenotypic and in vitro biochemical assays identified positions with a range of impacts on activity, including those that abolished resistance activity against tetracyclines, as well as those that retained resistance but had several-fold reductions in catalytic efficiency. Together, these results provide both a quantitative evaluation of FMO sequences for TDase activity, and a focused sequence-structure-function investigation of TDase enzymology.

## Results

### Development of gene models to predict new tetracycline-inactivating enzymes

**Fig. 1:**
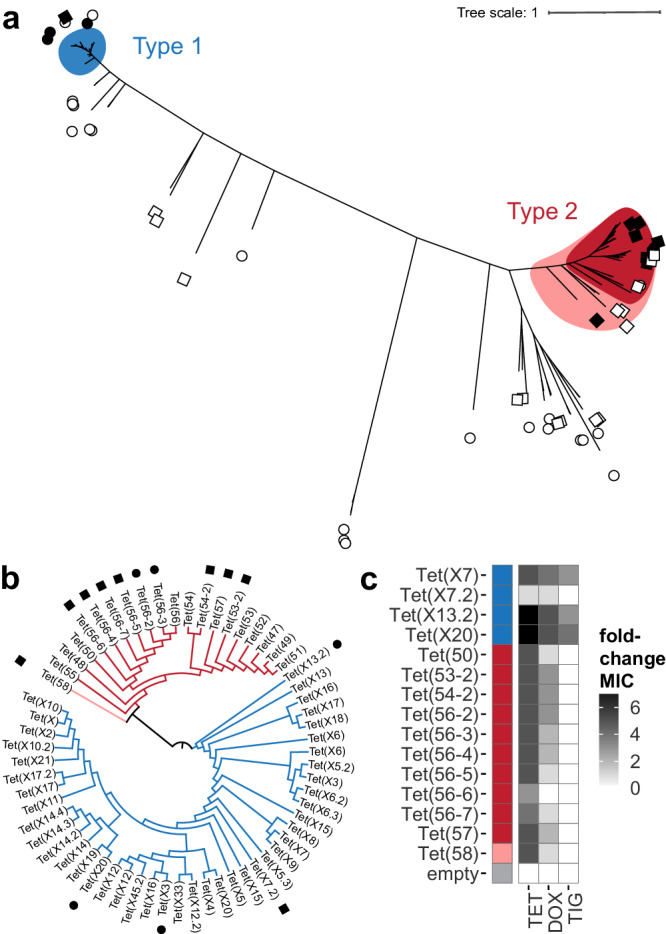
Characterization of novel TDases identified using profile HMMs. **a** Unrooted maximum-likelihood tree of tested HMM-predicted sequences and characterized TDase sequences. Generated with RAxML. Colored regions represent the type 1 clade (blue), the type 2 clade before HMM screening (red), and the expanded type 2 clade after HMM screening (pink). Circles indicate hmm01-predicted sequences: filled circle = functional (i.e. confers resistance to tetracycline); empty circle = does not confer resistance. Squares indicate hmm02-predicted sequences: filled square = functional; empty square = does not confer resistance. No circle or square = previously characterized TDase. **b** Maximum-likelihood cladogram of non-redundant characterized TDase sequences with branch lengths ignored. Generated with RAxML. Nodes annotated with name assigned in original report. Branch color indicates TDase clade (type 1 = blue; type 2 = red; type 2 after HMM screening = pink). Filled circles indicate functional sequences from hmm01, filled squares from hmm02. **c** Heatmap of MICs of newly identified TDase genes. Antimicrobial susceptibility testing was performed by microbroth dilution on sequences predicted by profile HMMs. Each box represents the consensus MIC value determined for three technical replicates, transformed as foldchange over the MIC values for the empty vector control. Colored box next to the protein names indicates the TDase type it belongs to (type 1 = blue; type 2 = red; type 2 after HMM screening = pink). Empty vector control, Tet(X7), and Tet(50) MICs included for reference. Acronyms = tetracycline (TET), doxycycline (DOX), tigecycline (TIG).

We predicted new TDase sequences by conducting a retrospective search of public sequence databases using profile-hidden Markov models (HMMs). We implemented this via a two-round process, where we performed a first round of HMM prediction followed by screening high-scoring sequences in *Escherichia coli* to determine if they confer tetracycline resistance. Then the positive results from this were used to build a second HMM that was used for a second round of prediction and screening. We generated the first HMM (hmm01) using published TDase amino acid sequences identified by functional metagenomics and the canonical Tet(X) (Supplementary Data 1)^7,21^. This was run on a combined protein sequence database that included the NCBI Refseq Non-redundant Protein Database^52^, the Human Microbiome Reference Genome Database^53^, the CARD database^54^, and sequences from functional metagenomic selections^55–63^. We then chose high-scoring sequences for synthesis and phenotypic testing in *Escherichia coli* (Fig. 1a). For this first round, we aimed to identify new TDase clades and thus prioritized sequences that were representative of the different major clades formed by phylogenetic comparison of high-scoring HMM hit sequences (Supplementary Fig. 2). We chose 18 candidate sequences outside of the canonical type 1 and 2 clades, with cutoffs at 40-80% amino acid identity to a characterized TDase sequence, and <92% amino acid identity (~30 amino acids different) to other candidate sequences. Additionally, we aimed to expand the diversity of the type 1 and 2 TDases, and thus also chose sequences that fell within these clades. We included 5 sequences from the type 1 clade with cutoffs at <99% amino acid identity (~5 amino acid different) to a known type 1 sequence and <92% amino acid identity to other candidate sequences, and 3 sequences within the type 2 clade with <92% amino acid identity to known type 2 sequences and <92% amino acid identity to other candidate sequences. We permitted testing of candidate sequences with relatively high similarity to known type 1 sequences because of the lower overall diversity of the type 1 clade. In total, for the first round of testing, we chose 26 sequences (5 type 1, 3 type 2, 18 outside) to be synthesized, cloned and inserted into a plasmid expression system, and transformed into *E. coli*. We recognized that heterologous expression in *E. coli* could result in false negatives due to various issues (e.g., codon preference, protein misfolding); however, previous successes using *E. coli* for production of recombinant TDases^7,21^ led us to conclude that it was the most appropriate host. Strains expressing these candidate HMM sequences were screened on 1 μg/mL tetracycline (the minimal inhibitory concentration, MIC, of the *E. coli* empty vector control) to determine if the sequences conferred a tetracycline resistance phenotype. A total of 5/26 cloned sequences resulted in resistance to tetracycline greater than the empty vector control confirming them as functional TDases (Fig. 1b). These were comprised of 3 type 1 sequences and 2 type 2 sequences. No sequences representative of clades outside the type 1 and 2 clades resulted in tetracycline resistance when expressed in *E. coli*.

For the second round, we constructed an HMM (hmm02) using all sequences used to generate the first HMM, plus the 5 new TDase sequences discovered from the hmm01 screening, as well as 9 additional characterized sequences from the literature (Supplementary Data 1). In this round, in addition to a combined HMM using sequences from both types, we also generated separate HMMs specific for the type 1 and type 2 TDases. These second-round HMMs were then run against the same combined sequence database, as well as sequences from metagenome-assembled genomes^64–69^, and NCBI BLAST hits from Tet(50) with >35% amino acid identity to this sequence. In contrast to the first round’s goal of broadly screening for new TDase clades, here we aimed to expand the diversity of known clades. We focused particular attention on the type 2 clade because it has greater intra-clade diversity than the type 1 clade and fewer known sequences. Therefore, we chose 13 sequences that fell within the type 2 clade. Additionally, among the 10 sequences outside of the type 1 and 2 clades, we included 4 sequences on branches adjacent to the type 2 clade with ~60% amino acid identity to a type 2 sequence. In this round, we chose only 1 sequence within the type 1 clade. The same amino acid identity cutoffs used in the first-round screening were also applied here. In total, for the second round of testing we chose 24 sequences (1 type 1, 13 type 2, 10 outside) to be synthesized and cloned into a plasmid expression system, and transformed into *E. coli*. Screening on tetracycline revealed 9/24 cloned sequences as functional TDases (Fig. 1a, b). These were comprised of 1 type 1 sequence, 7 type 2 sequences, and 1 sequence that fell outside of the type 2 clade, but its confirmed activity and relative similarity (55% amino acid identity) represent an expansion of the diversity of this clade.

Since the general selection criteria for choosing sequences for functional testing in the two HMM rounds were different—low identity exploration of new clades via hmm01 vs higher identity expansion of known clades via hmm02—it is not appropriate to compare the functional validation rate of the two rounds. Instead, we used the combined functional testing results to retrospectively identify type-specific HMM sequence bit score thresholds (using the second-round HMMs) that optimally discriminate between functional and nonfunctional sequences from our screening. The sequence bit score is the log-odds score of a target sequence against a given HMM, and is what the sequence E-value is based on ref. ^70^. Sequence bit score was used here instead of E-value because the bit score does not depend on the size of the sequence database. A bit score of 455 with the combined HMM had a specificity of 0.81 and sensitivity of 1.0. For sequences with >40% amino acid identity to a type 1 sequence a bit score of 792 from the type 1-specific HMM had a sensitivity and specificity of 1.0. Lastly, for sequences with >40% amino acid identity to a type 2 sequence, a bit score of 661 with the type 2-specific HMM had a specificity of 0.91 and sensitivity of 0.91 (Supplementary Fig. 2).

### Characterization of new TDases

In total, tetracycline screening validated 14/50 of our candidate HMM sequences as TDases, comprising 4 type 1 sequences and 10 type 2 sequences (Fig. 1a, b; Supplementary Data 1). We assigned these newly identified sequences *tet* names based on published tetracycline resistance gene naming conventions (Methods), which classified them as: 2 new *tet* genes, 8 *tet* gene variants, 1 Tet(X) gene subvariant, 2 Tet(X) gene variants that had been previously reported but with unconfirmed activity, and 1 reidentified sequence that has been previously reported and characterized as Tet(X3)^29^. The Tet(X3) sequence was identified during the screening of hits from hmm01, which was not constructed using the Tet(X3) sequence. The two new *tet* genes are designated Tet(57) and Tet(58), and the closest sequences to them are Tet(53) and Tet(55) with 76.2% and 55.0% amino acid identity, respectively. Of the 8 type 2 *tet* gene variants we identified 6 sequences with 81.4 to 87.9% amino acid identity to Tet(56) and designated these as Tet(56)-2 to -7. We also identified one sequence with 82.2% amino acid identity to Tet(53) and designated it Tet(53-2), and one with 87.9% amino acid identity to Tet(54) and named it Tet(54-2). We identified one type 1 TDase subvariant with 91.0% amino acid identity to Tet(X7) and named this Tet(X7.2). The remaining two sequences had 100% identity to the sequences previously named Tet(X20) and Tet(X10) by Umar et al. ^71^; however, the functionality of these sequences had not previously been tested and were named based on sequence similarity alone. Therefore, our analysis represents the first experimental characterization of these sequences. We retained the sequence name Tet(X20), but because Tet(X10) had already been assigned to another characterized sequence^21^ we instead named it Tet(X13.2) because it has 99.5% amino acid identity to Tet(X13).

These *E. coli* + pZE24 strains expressing positive hit sequences were further characterized by antibiotic susceptibility testing (AST) against doxycycline (second-generation tetracycline) and tigecycline (third-generation tetracycline; Fig. 1c). Consistent with previous studies, sequences in the type 1 clade conferred resistance against all generations of tetracyclines, while the type 2 clade conferred resistance against first- and second-generation tetracyclines but not the third-generation tigecycline.

We also synthesized, cloned, and functionally tested other sequences previously described in the literature as tetracycline-inactivating enzymes that fall outside of type 1 and 2 framework: MabTetX^72^, Tet37^73^, Tet(X1) with an N-terminal extension that was missing in the original annotation^74^, and two sequences predicted by structure-based HMMs (MC3.MG14.AS1.GP1.C5528.G2 and MC3.MG338.AS1.GP1.C21977.G2)^75^. These other sequences are highly dissimilar to known TDases. Tet(X1) is most similar to Tet(X12) at 69.6% amino acid identity; however, all the other sequences have <25% amino acid identity to a known TDase. They all also had sequence bit scores well below the cutoff for the combined HMM, and Tet(X1) was also below the cutoff for the type 1-specific HMM with a sequence score of 634. Based on these metrics we predicted they would not be functional, and indeed none of these sequences conferred resistance to *E. coli* in our expression system (Supplementary Data 1). Notably, these results are consistent with previous reports that also did not detect tetracycline resistance from MabTetX or Tet(X1) cloned into *E. coli*^74,76^; however, the observed lack of functionality may be due to problems with recombinant expression or protein folding in *E. coli*.

### Kinetics of the enzymatic inactivation of tetracycline

**Fig. 2:**
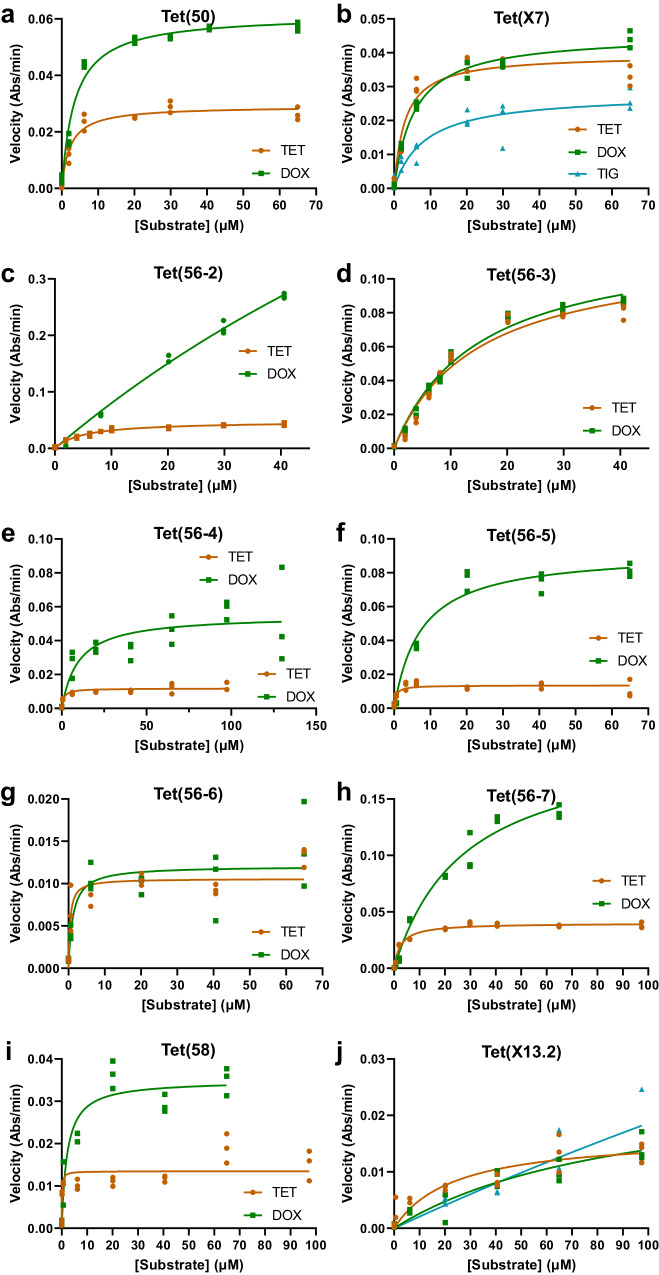
Steady-state kinetic plots of tetracycline inactivation by HMM-identified enzymes. Steady-state kinetic plots of velocity vs substrate concentrations for the TDase-catalyzed degradation of tetracycline antibiotics were fit to the Michaelis-Menten equation. The previously characterized TDases (**a**) Tet(50) and (**b**) Tet(X7) were recharacterized here as reference to the newly identified type 2 TDases (**c**) Tet(56-2), (**d**) Tet(56-3), (**e**) Tet(56-4), (**f**) Tet(56-5), (**g**) Tet(56-6), (**h**) Tet(56-7), and (**i**) Tet(58), as well as the new type 1 TDase (**j**) Tet(X13.2). Acronyms = tetracycline (TET), doxycycline (DOX), and tigecycline (TIG). Error values represent the standard deviations for three independent trials.

We next tested the in vitro degradation of tetracycline, doxycycline, and tigecycline by eight of the new, phenotypically validated TDases by expressing and purifying recombinant *N-*His_6_-tagged enzymes (Fig. 2). To understand substrate binding and catalytic efficiency for drug inactivation we measured apparent Michaelis-Menten kinetic parameters and compared these to the previously determined parameters of Tet(X7) and Tet(50) (Fig. 2a, b; Supplementary Data 1). While the apparent catalytic efficiencies (*k*_*app*_*/K*_*M*_) of Tet(56-2) and Tet(56-3) are similar to Tet(50) for the inactivation of tetracycline and doxycycline, the *K*_*M*_ and *k*_*app*_ for the inactivation of doxycycline by Tet(56-2) are 150 ± 42 µM and 3.2 ± 0.2 min^−1^ respectively (Fig. 2c-d). This is much larger than that of Tet(50) (*K*_*M*_ and *k*_*app*_ values of 3.57 ± 0.44 μM and 0.15 ± 0.02 min^−1^ respectively), suggesting that while Tet(56-2) has a much lower binding affinity for doxycycline, the turnover rate is higher. Additionally, the apparent catalytic efficiency of Tet(58) was 8 times greater than that of Tet(50) for the degradation of tetracycline (*k*_*app*_*/K*_*M*_ values of 0.28 ± 0.650 and 0.03 ± 0.024 μM^−1^ min^−1^, respectively (Fig. 2i). This difference in apparent catalytic efficiencies is largely mediated by Tet(58)’s high affinity for tetracycline substrate (i.e. lower *K*_*M*_) as the two enzymes’ *k*_*app*_ values are of similar magnitude; however, we note that the curve fit was low (R-squared = 0.691) in this case. As shown previously^77^, the type 1 and 2 enzymes overall have similar catalytic efficiencies at degrading first- and second-generation tetracyclines (TET and DOX, respectively).

### Determining X-ray crystal structures of new TDases from *Legionella* spp

**Fig. 3:**
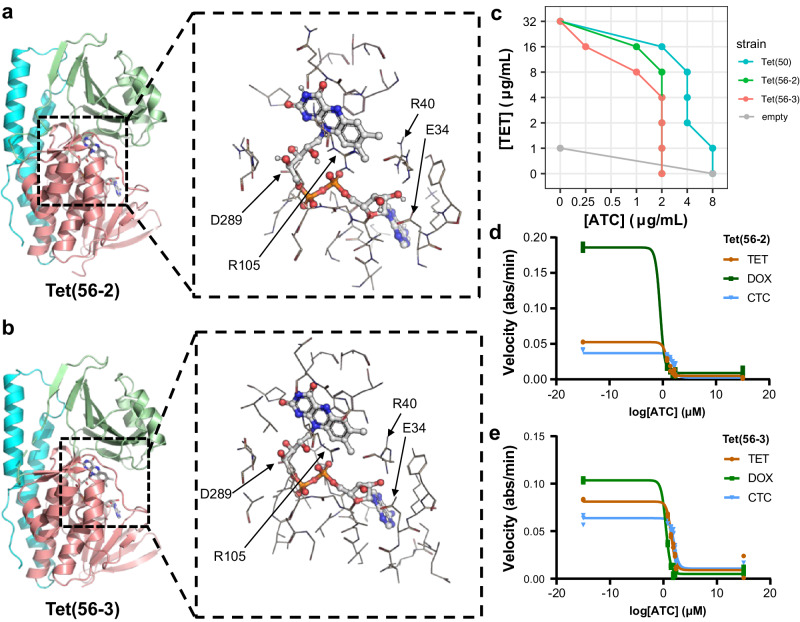
X-ray structures and inhibition of type 2 TDases from *Legionella* spp. X-ray structures of (**a**) Tet(56-2) (PDB 8TWG) and (**b**) Tet(56-3) (PDB 8TWF). The FAD-binding domain (FBD) is colored salmon, the substrate-binding domain (SBD) is colored pale green, and the C-terminal helix is colored cyan. Residues within 5 Å sphere around the bound co-factor, FAD, are represented as lines. Key residues that form H-bonds are highlighted for each structure. The substrate-binding domain is colored pink, the FAD-binding domain is colored orange, the C-terminal bridge helix is colored blue, and the additional C-terminal helix specific to type 2 TDases is colored purple. Inset = Key residues that interact with FAD and are located in the substrate binding pocket are highlighted. **c** Checkerboard whole cell inhibition of *E. coli* expressing TDases by anhydrotetracycline. Each point indicating the concentration of anhydrotetracycline that lowers the MIC of each strain to a given concentration of tetracycline. **d** In vitro anhydrotetracycline inhibition of Tet(56-2) and (**e**) Tet(56-3) degradation of tetracycline antibiotics visualized as apparent IC_50_ curves generated using enzyme velocities measured via an optical absorbance assay. Error values represent the standard deviations for three independent trials. Acronyms = tetracycline (TET), doxycycline (DOX), chlortetracycline (CTC), and anhydrotetracycline (ATC).

We determined the X-ray structures of Tet(56-2) (PDB 8TWG) and Tet(56-3) (PDB 8TWF), two new TDases discovered in this study. Tet(56-2) and Tet(56-3) are most closely related to Tet(56), at 87.4% and 87.9% amino acid identity respectively, and have 88.6% amino acid identity to each other. Tet(56) is the only type 2 TDase identified to date in a pathogenic bacterium, *Legionella longbeachae*. Tet(56-2) and Tet(56-3) were also identified in the genomes of *Legionella* species: *L. clemsonensis* and *L. massiliensis*, respectively. We determined the X-ray structures of Tet(56-2) at 1.80 resolution with Rfree/Rwork of 17.43/20.93 and Tet(56-3) at 2.39 Å with Rfree/Rwork of 21.69/24.85 (Table 1). Both structures exhibit the conserved architecture of type 2 TDases, consisting of a FAD-binding Rossman fold domain, a tetracycline-binding domain, a C-terminal bridge helix that links the two domains, and a unique C-terminal helix, known as the “gate-keeper helix” (Fig. 3a, b).

**Table 1:** Data reduction statistics for the Tet(56-2) and Tet(56-3) structures

	Tet(56-2)	Tet(56-3)
*Data collection*		
Space group	P 2 2_1_ 2_1_	P 2_1_ 2_1_ 2_1_
*Cell dimensions*		
*a*, *b*, *c* (Å)	46.79 70.55 128.31	77.79 133.98 164.63
a, b, g (°)	90.00 90.00 90.00	90.00 90.00 90.00
Resolution (Å)	19.97–1.80 (1.86–1.80)	19.74–2.39 (2.52–2.39)
*R*_meas_	0.07 (0.54)	0.09 (0.53)
I /s	17.78 (3.34)	15.07 (3.27)
CC(1/2)	99.9 (93.5)	99.9 (87.3)
Completeness (%)	99.6 (99.0)	99.6 (99.0)
Redundancy	6.51 (6.36)	6.57 (6.37)
*Refinement*		
Resolution (Å)	19.97 – 1.80	19.74 – 2.40
No. reflections	40,127	68,297
Rwork / Rfree	17.43/20.93	21.69/24.85
*No. atoms*		
Protein	3073	12,041
Ligand	84	336
Water	259	159
*B-factors*		
Protein	29.86	45.68
Ligand	23.71	33.39
Water	33.00	37.97
*R.m.s. deviations*		
Bond lengths (Å)	0.009	0.003
Bond angles (°)	0.88	0.52
*Validation*		
MolProbity score	1	1.2
Clashscore	1.45	1.45
Poor rotamers (%)	0	0
*Ramachandran plot*		
Favored (%)	97.39	95.27
Allowed (%)	2.61	4.73
Disallowed (%)	0	0

Each structure was solved from a single crystal. Values in parentheses are for highest resolution shell.

The FAD cofactor adopts a seemingly conserved “OUT” conformation in both Tet(56-2) and Tet(56-3) substrate-free structures, consistent with the “waving” flavin phenomenon previously described in other type 2 TDases^23^. When the FAD cofactor adopts the “OUT” conformation it is exposed to solvent and presumed to be reduced by NADPH. The reduced FAD is then presumed to transition to the “IN” conformation where it moves to the active site to be oxidized by O_2_ to form the reactive FAD-C4a-peroxy flavin species. While in the active site adopting the “IN” conformation, the TDase facilitates hydroxyl group transfer from the FAD-C4a-peroxy flavin to the bound tetracycline substrate. Additionally, we observed that the loop containing F95 shows a distinct conformation in the Tet(56-2) structure, in which F95 is oriented away from the substrate-binding channel, thus presenting the loop with an opening for substrate to enter the active site (Supplementary Fig. 3). The region containing F95 was disordered in the previously solved Tet(56) structure (PDB 5TUM) and thus could play an important role in active site gating^23^. Most residues in the FAD-binding pocket and the substrate/inhibitor binding pocket are conserved across the three *Legionella* Tet(56) structures. The “gate-keeper” C-terminal helix of Tet(56-2) and (Tet56-3) orients similarly to the previously solved Tet(56) structure but differently from the previously solved Tet(50) structure (Supplementary Fig. 3)^23^.

### Anhydrotetracycline can rescue tetracycline inactivation

We previously established that anhydrotetracycline is a pan-TDase inhibitor^16,23,77,78^. Here we sought to study whether anhydrotetracycline can rescue tetracycline activity against *E. coli* producing Tet(56-2) and Tet(56-3). We identified anhydrotetracycline concentrations that result in a tetracycline MIC less than that of tetracycline alone using checkerboard broth microdilution antibiotic susceptibility assays that test for cell growth in multiple tetracycline-anhydrotetracycline combinations. The addition of 1 μg/mL anhydrotetracycline reduced the concentration of tetracycline required to inhibit the growth of Tet(56-2) producing *E. coli* two-fold, from 32 μg/mL to 16 μg/mL (Fig. 3c). Similarly, the addition of 1 μg/mL anhydrotetracycline reduced the concentration of tetracycline required to inhibit the growth of Tet(56-3) producing *E. coli* four-fold, from 32 μg/mL to 8 μg/mL (Fig. 3c). While anhydrotetracycline has antibiotic activity on its own against *E. coli*, we tested for inhibition at concentrations below the anhydrotetracycline-alone MICs for strains expressing Tet(56-2) and Tet(56-3) (2 μg/mL). Notably, the anhydrotetracycline-alone MICs for *E. coli* expressing Tet(56-2) and Tet(56-3) were four-fold lower than those for the same strain expressing Tet(50) (8 μg/mL).

Lastly, we evaluated the inhibitory activity of anhydrotetracycline against Tet(56-2)- and Tet(56-3)-mediated degradation of tetracycline antibiotics (Fig. 3d; Supplementary Data 1). The apparent half-maximal inhibitory concentrations (IC_50_) were higher for the inhibition of chlortetracycline degradation for both enzymes compared to tetracycline and doxycycline. This trend has been previously observed for the anhydrotetracycline inhibition of Tet(X) and Tet(X7)^77^. Apparent IC_50_ values for anhydrotetracycline inhibition of Tet(56-2) and Tet(56-3) were in the low micromolar range (0.3-30 µM) for tetracycline and doxycycline inhibition. Together these results suggest that anhydrotetracycline inhibition is a promising combination therapy against bacteria producing Tet(56-2) and Tet(56-3).

### Identification of amino acid positions conserved across the entire TDase family

**Fig. 4:**
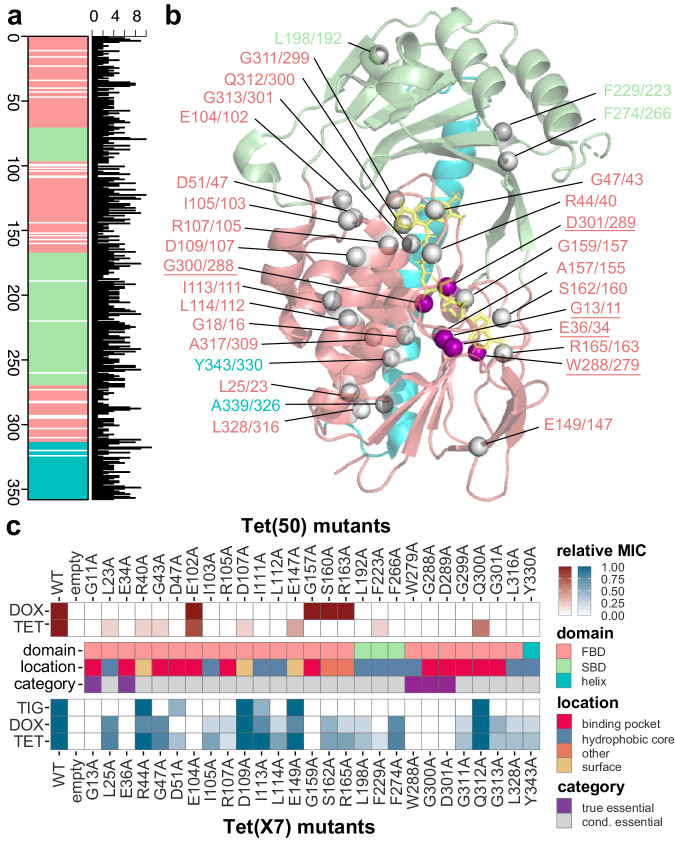
Identification of amino acid positions essential to TDase function. **a** Linear geneplot of TDase combined MSA. White lines indicate a 100% conserved position (i.e. only 1 amino acid is observed in the MSA). Colored regions indicate the FAD-binding domain (FBD; salmon), substrate-binding domain (SBD; pale green), and the C-terminal helix (cyan). Adjacent barplot indicates the number of different amino acids observed at a given position. **b** The Tet(X7) monomer A (6WG9) crystal structures with the 31 conserved residues shown as spheres at C alpha positions. Residues are labeled with the Tet(X7) sequence position first followed by the Tet(50) sequence position, and colored according to the domain the residue is in. True essential residues as determined by AST testing alanine-scanning mutants are underlined and the spheres are colored purple, while conditionally essential residues are colored gray. **c** Heatmap of the impact of alanine substitutions on resistance activity. Top (red) = Tet(50) mutants; Bottom (blue) = Tet(X7) mutants; Middle indicates the domain and location where the residues are found, and what AST results categorized them as based on resistance activity. MICs were transformed as the foldchange value relative to the wild-type and empty vector negative control strains’ MICs, where 1.00 (dark blue or dark red) = MIC equivalent to wild-type strain, and 0.00 (white) = MIC equal to the empty vector (i.e. no activity). Acronyms = tetracycline (TET), doxycycline (DOX), and tigecycline (TIG).

Residues critical for enzyme function are conserved across protein families, as their substitution results in loss of function (e.g., impaired folding, decreased stability, poor substrate or cofactor binding)^42,43^. The profile HMMs described above work by capturing position-specific statistical probabilities for how conserved each amino acid is at each position of a multiple sequence alignment (MSA), and query sequences which have a different residue than what is observed in the underlying MSA are penalized. To comprehensively identify conserved amino acid residues, we analyzed 114 functional, non-redundant TDase sequences that have been verified as tetracycline resistance genes through recombinant expression, isolate screening, and/or functional metagenomic selections, including the 13 new TDases we identified by HMM screening in this work (Fig. 1a–c)^7,8,20–22,26,27,29–34,37,38,71,79–82^. These 114 sequences were used to generate MSAs specific to type 1 and type 2 TDases (comprised of 91 and 23 sequences, respectively), and a combined MSA of both types (114 sequences). The type 1 MSA had 238 positions conserved across all sequences in the MSA, the type 2 MSA had 76 conserved positions, and the combined type 1 + 2 MSA had 31 conserved positions (Fig. 4a; Supplementary Fig. 4). The greater number of conserved positions in the type 1 MSA is reflective of the fact that, despite having nearly 4x more functional sequences discovered than the type 2 clade, the overall diversity of the type 1 TDases is lower than that of the type 2 s (type 1 median pairwise amino acid identity = 90.8%; type 2 median pairwise amino acid identity = 68.2%). To identify residues essential for tetracycline-inactivating activity generally, we focused further analyses on the combined MSA.

By mapping the 31 conserved positions onto the crystal structures of the type 1 TDase Tet(X7) and the type 2 TDase Tet(50), we inferred that most have functional roles in substrate binding, cofactor binding, and structural stabilization (Fig. 4b; Supplementary Data 1). The structural location of equivalent conserved residues in Tet(X7) and Tet(50) are in approximately the same location in both enzymes, consistent with the similarity of their three-dimensional structures despite low sequence identity (Supplementary Fig. 5a)^23^. Since equivalent amino acid residues between these two proteins (i.e. same column in MSA, same 3D position) may have different sequence position numbers due to insertions/deletions throughout the length of the sequence, we refer to equivalent positions in both proteins as: the amino acid, the position number in Tet(X7), a slash, and the position number in Tet(50). For example, “G13/11” refers to G13 in Tet(X7) and G11 in Tet(50) which are equivalent positions despite different numbering. Most conserved residues were located in the FAD-binding domain (FBD; 26/31), with relatively few in the substrate-binding domain (SBD; 3/31) and the C-terminal helix (2/31) (Fig. 4a, b). Additionally, most were located in the binding pocket (14/31) or the hydrophobic core (12/31), with the remaining residues being on the surface (3/31) or forming hydrogen bonds with neighboring residues presumably to stabilize the folded state (2/31) (Supplementary Fig. 5b–d; Supplementary Data 1). Conserved residues include those in the canonical GxGxxG motif associated with binding to the flavin adenine dinucleotide (FAD) cofactor, a feature that is found across the entire FMO family (Tet(X7)/Tet(50) positions: G13/11, G18/16)^83^. They also include positions previously identified in the type 1 TDases Tet(X) and Tet(X7) as FAD-binding (E36/34, G47/43) or substrate-binding (G311/299)^21^, suggesting that the functional role of these positions extends to all type 1 and 2 TDases.

### Alanine-scanning mutagenesis phenotypically validates the role of conserved positions

To evaluate the role of these conserved residues, we generated site-directed alanine scanning mutants of the type 1 TDase Tet(X7) and the type 2 TDase Tet(50) at 27 conserved positions (54 mutants total) identified in the combined MSA (Supplementary Data 1). We predicted that mutations at most conserved residues would result in loss of function indicated by decreased MICs, and fall into one of four categories based on the impact of that substitution: (1) “true essential” residues would have a complete loss of resistance activity to all antibiotics in both enzymes, (2) “conditionally essential” residues would have a complete loss of activity to at least one antibiotic in at least one enzyme, (3) “important” residues would maintain resistance to all drugs in both enzymes but with reduced MICs and/or biochemical kinetics, and (4) “false positive” residues would retain activity equivalent to wild-type. We did not expect any mutations would increase activity relative to wild-type, as mutations at critical sites would be expected only to have negative or neutral impacts.

We observed that alanine substitutions at all tested positions had a range of negative impacts on resistance to tetracycline antibiotics, thus classifying them as either true or conditionally essential. We performed ASTs on *E. coli* expressing these 54 mutant proteins against tetracycline, doxycycline, and tigecycline (Fig. 4c; Supplementary Data 1). We observed 5/27 mutated residues were “true essential” residues as their substitution resulted in the complete abolishment of resistance activity across all antibiotics tested in both enzymes at—G13/11, E36/34, W288/279, G300/288, and D301/289. All five were located in the FBD, with 4/5 located in the FAD-binding pocket, suggesting the loss of activity is due to mutations interfering with the ability to interact with the FAD cofactor (Supplementary Fig. 5b, c). The last residue in this category (W288/279) is located in the hydrophobic core, suggesting activity loss could be due to these mutants being unable to properly fold. The remaining 22/27 positions were “conditionally essential,” with complete loss of resistance activity to at least one drug in at least one enzyme (Fig. 4c). Importantly, we did not observe false positives across TDase mutants from both types, validating this approach for identifying amino acid residues with important functional roles.

We observed variability within the conditionally essential category depending on the enzyme background in which the substitution was evaluated. Most Tet(50) mutants completely lost resistance to tetracycline and doxycycline, but activity against these drugs in equivalent Tet(X7) mutants was retained, albeit with reduced MICs (Fig. 4c). This was unexpected, as the greater intra-clade sequence diversity of type 2 enzymes like Tet(50) suggested these would be more accommodating of substitution than the type 1 enzymes. And yet, it appears that the type 1 sequences like Tet(X7) are more resilient to loss of function mutations even when substitutions occur at these conserved sites important for function. For five positions—R44/40, D109/107, I113/111, E149/147, and Q312/300—the Tet(X7) mutants maintained at or near wild-type activity levels for all antibiotics tested, while the equivalent positions in Tet(50) had complete loss of doxycycline activity and greatly reduced tetracycline activity. All of these amino acid residues are located in the FBD with 3/5 located on the protein surface. The opposite, where Tet(50) maintained near wild-type activity while Tet(X7) completely lost activity, was seen at just one position—E104/102, also located in the FBD but in the binding pocket (Fig. 3c). Mutations in Tet(X7) impacted tigecycline resistance more than tetracycline or doxycycline resistance at 13/31 positions (L25, G47, I105, R107, L114, S162, R165, L198, F274, G311, G313, L328, and Y343). This could be a reflection of the extended interactions and limited substrate binding modes that are accessible to the glycylcyclines like tigecycline that require the extension of the sterically bulky D-ring substituents to the solvent-exposed side of the active site^27,84^.

We found that true essential residues are conserved across class A FMO sequences, indicating they are important for TDases’ function as FMOs. In order to discriminate between residues important for TDases’ function as tetracycline-inactivating enzymes versus more broadly as FMOs, we generated an MSA of all 114 TDase sequences plus 14 FMO sequences representative of the class A FMO family^85^. In this MSA, five residues were conserved across both TDases and FMOs, suggesting they are important specifically for FMO functionality: G13/11, G18/16, G159/157, G300/288, and G301/289. Nearly all of these were classified via our alanine-scanning mutagenesis in TDases as true essential residues (Fig. 4c). The remaining conserved true essential residues, E36/34 and W288/279, are conserved in a subset of the FMO sequences, again suggesting they are essential to TDases’ activity as FMOs. Lastly, we used this MSA to identify conserved residues which characterize TDases apart from the broader FMO family. We found that D51/47 and E104/102 are conserved in all TDases but different in the other FMOs. Both residues are located in the FAD-binding pocket in close proximity to each other (Supplementary Fig. 5).

### Mutants which retained resistance had large reductions to catalytic efficiency, but showed limited change in thermal stability

**Fig. 5:**
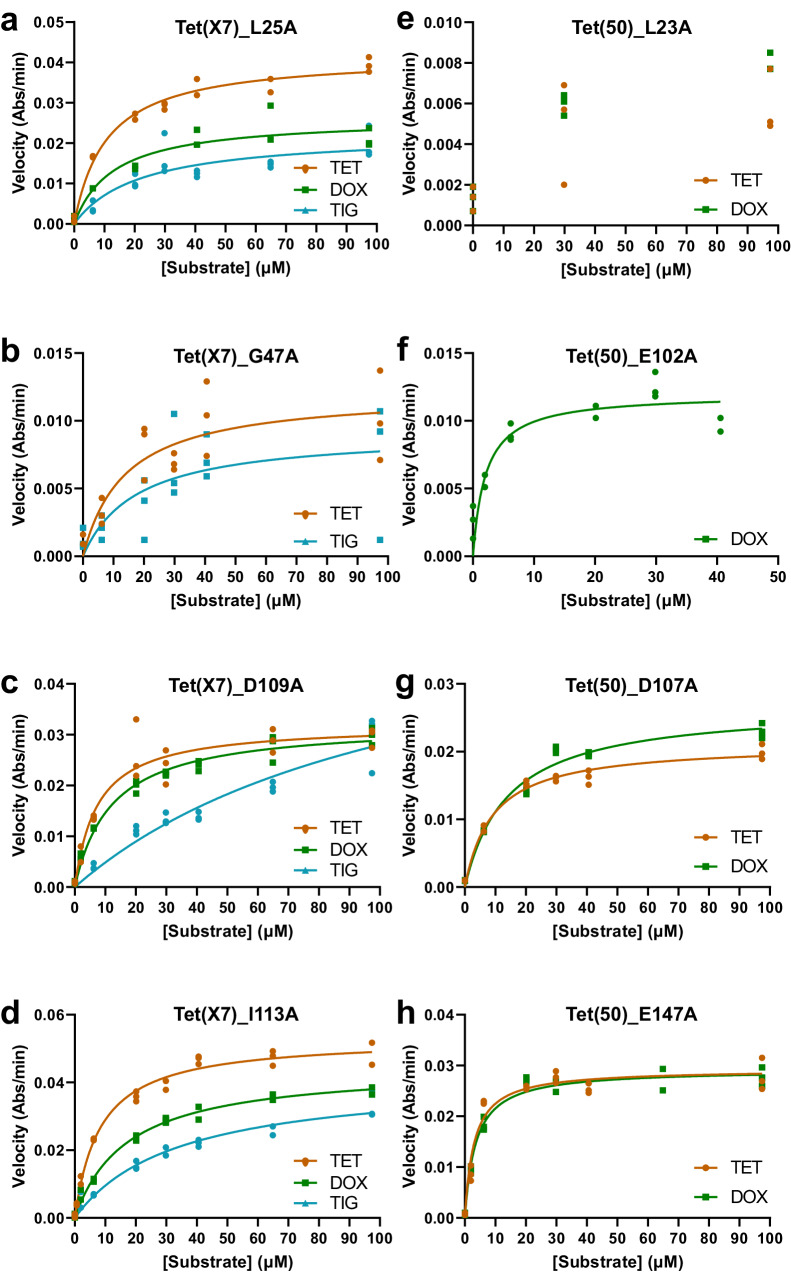
Steady-state kinetic plots of tetracycline inactivation by alanine-scanning mutant enzymes. Steady-state kinetic plots of velocity vs substrate concentrations for the TDase-catalyzed degradation of tetracycline antibiotics were fit to the Michaelis-Menten equation. The degradation rates of tetracycline, doxycycline, and tigecycline were measured for the following TDase alanine-scanning mutants: Tet(X7) mutants (**a**) L25A, (**b**) G47A, (**c**) D109A, and (**d**) I113A, and the Tet(50) mutants (**e**) L23A, (**f**) E102A, (**g**) D107A, (**h**) E147A. See Fig. 2a, b for wild-type Tet(X7) and Tet(50) plots, for reference. Acronyms = tetracycline (TET), doxycycline (DOX), and tigecycline (TIG). Error values represent the standard deviations for three independent trials.

Alanine-scanning mutants which retained near wild-type MICs to some tetracycline antibiotics nevertheless lost function in terms of biochemical kinetics, indicated by substantial reductions to in vitro catalytic efficiency (*k*_*app*_*/K*_*M*_) during degradation of those tetracycline antibiotics (Fig. 5; Supplementary Data 1). For example, Tet(X7)_D109A and Tet(50)_D107A were mutated at equivalent positions at the surface of each protein, but while the Tet(X7) mutant retained full MICs for all drugs, the Tet(50) mutant lost doxycycline resistance and showed a 16-fold decrease in tetracycline resistance (20% of the WT MIC). Despite these differences, both mutants exhibited roughly equivalent reductions in tetracycline and doxycycline kinetics, falling to 10-20% of the WT *k*_*app*_*/K*_*M*_. This was driven by an increase in *K*_*M*_ indicating lower affinity for the substrate. Inversely, Tet(X7)_E104A and Tet(50)_E102A were mutated at equivalent positions located in the FAD-binding pocket close to the flavin ring, but while the Tet(50) mutant had 80-100% the WT MIC to tetracycline and doxycycline the Tet(X7) mutant completely lost resistance. However, the Tet(50) mutant had ~30% catalytic efficiency compared to the WT enzyme. This was driven by an apparent 5-fold decrease in *k*_*app*_ indicating reduced catalytic efficiency relative to WT Tet(50) (Supplementary Data 1). Lastly, the mutants Tet(X7)_L25A and Tet(X7)_I113A retained tetracycline and doxycycline MICs at 80-100% of WT activity, while substitution at equivalent positions in Tet(50) resulted in complete or near-complete loss for both drugs. However, these Tet(X7) mutants’ *k*_*app*_*/K*_*M*_ to these drugs was 30-50% of WT activity.

To determine if these single amino acid substitutions influenced stability, we measured melting temperatures for a variety of type 1 and 2 TDase mutants. We observed that most of the mutant proteins retained a melting temperature approximately the same as that of the WT proteins (Supplementary Fig. 6). The WT Tet(X7) protein had an apparent T_m_ of 43 °C, and WT Tet(50) had an apparent T_m_ of 49 °C. Most mutants had melting temperatures approximately ±2 °C of their WT counterpart. The exceptions are Tet(50)_S160A, which exhibited an apparent increase in thermal stability of 8 °C to 57 °C due to a mutation in a stabilizing loop. However, this increased stability came at the cost of complete loss of resistance against tetracycline. The largest reduction in stability was observed in Tet(X7)_G47A, which decreased by 4 °C to 39 °C due to a mutation in the FAD-binding pocket. This mutant retained MIC 80% of WT for tetracycline but had only 10% catalytic efficiency (*k*_*app*_*/K*_*M*_) of WT Tet(X7). This was driven by an apparent increase in *K*_*M*_ indicating a reduced binding affinity for the substrate, which is consistent with this position’s previously described role as a FAD-binding residue. Overall, there was no apparent correlation between thermal stability and resistance phenotypes for the mutants we tested. The steady-state kinetic studies support a model where the substitutions studied here influence *k*_*app*_ and/or *K*_*M*_ as the driver of the resistance phenotype whereas their thermal stabilities are largely unaffected.

Lastly, bringing together the HMM sequence identification and MSA sequence determinants of function, we examined whether non-functional HMM hit sequences within the type 1 and 2 clades could be explained by mutations at conserved sites. A total of 6/7 non-functional HMM hit sequences in the expanded type 2 clade (Fig. 1a) had different residues at one or more of the following conserved sites, which when mutated to alanine in Tet(50) resulted in a complete loss of tetracycline activity (i.e., at least conditionally essential): I103, I111, L112, E147, L192, and/or F266 (Fig. 4c). However, we note that we do not have the phenotypic data to state whether residues other than alanine at these conserved positions would also abolish resistance activity, or whether one or more mutations at non-conserved sites could do the same (indeed, this must be the case for the seventh non-functional type 2 clade sequence which did not have substitutions at any of the conserved residues). Similarly, two non-functional HMM hit sequences in the type 1 clade had substitutions at the conserved sites D51 or R165; however, sequence comparisons later showed were not full-length sequences (i.e. N- and C-terminal truncations) and we attribute the lack of functionality to this rather than the D51 or R165 substitutions.

## Discussion

In this study, we used complementary sequence-based approaches to discover new TDase variants and gain insight into the sequence determinants of their tetracycline-inactivating function. Using profile-hidden Markov models (HMMs), we identified 50 high-scoring FMO sequences from publicly available sequence databases. We cloned these sequences into an expression system in *E coli*, and screened the resultant transformants for functional inactivation of tetracycline, leading to the discovery of 13 new TDases. Using a MSA-based approach, we identified amino acid positions conserved across 114 functionally validated TDase sequences, and evaluated the role of these important positions by generating 54 alanine-scanning mutants in Tet(X7) and Tet(50).

A similar HMM-based approach was recently reported by Berglund et al. ^34^, which tested 5 TDase-like hits and confirmed one as functional. One of our aims in using this approach was to identify cryptic clades of TDases (i.e., type 3); however, none of the tested HMM hits with sufficiently low amino acid similarities known TDases to qualify for that conferred tetracycline resistance. This may reflect insufficient sampling and screening, and/or sequence incompatibilities with our *E. coli* heterologous expression system. Indeed, a MabTetX sequence has been reported as a functional tetracycline-inactivating enzyme in *Mycobacterium spp*^72^, and its low identity to either the type 1 or 2 clades ( < 25% amino acid identity) would warrant classification as a “type 3” TDase. However, this sequence does not confer tetracycline resistance in *E. coli*. Notably, this limitation will only result in false negatives and does not impact the utility of true positive sequences identified and characterized using this approach.

Most type 2 TDase sequences thus far have been identified only through functional metagenomic selections, limiting analyses into host background beyond association with soil microbiomes. Here, by scanning public sequence databases that include draft genomes of cultured bacteria, we can begin to connect sequences with species. For example, Tet(56) was identified in *Legionella longbeachae*, and several of our newly identified variants of Tet(56) were identified in *Legionella* spp. genomes: Tet(56-2) in *L. clemsonensis*, Tet(56-3) in *L. massiliensis*, Tet(56-5) in *L. busanensis*, and Tet(56-7) in *L. feeleii*. This suggests that these sequence variants may have diverged along with *Legionella* during speciation. However, when comparing the genomic regions surrounding these sequences we did not observe similarity or synteny, nor did we observe the presence of insertion sequences that might suggest horizontal transfer. Further work into the evolution of these sequences within *Legionella* and other taxonomic groups is warranted.

The structural findings from Tet(56-2) and Tet(56-3) provide insights into the conserved mechanism of tetracycline inactivation in type 2 TDases and reveal important differences from other type 2 TDases. The structures of Tet(56-2) and Tet(56-3) show that the FAD binds in an “OUT” conformation, a conformation that was previously confirmed in X-ray structures of other type 2 TDases. The FAD-interacting residues are conserved between both structures. Intriguingly, L181 and T183 in *Legionella* Tet(56) enzymes are replaced with V181 and A183 in Tet(50). Such mutations merit further study to determine if there is a correlation with improved catalytic efficiency and/or resistance phenotype. Sequence alignment of type 2 TDases indicates that the region encompassing D356 in Tet(50) is variable, and some type 2 TDases contain a gap in this region.

With multiple groups publishing new TDase sequences simultaneously without a central classification scheme, the field contains many naming inconsistencies. Different sequences have been reported under the same name, the same sequence has been given different names, and sequences have been named without experimental confirmation of activity. We have chosen to label previously reported sequences with the name assigned in the paper it was first reported in, even if that is in conflict with another sequence. For clarity, we also included full amino acid sequences and GenBank accession numbers (Supplementary Data 1). However, we echo the conclusions of previous reports in stating that the field should adopt a consistent classification scheme^86^, which requires newly named Tet(X) variants have: i) experimental confirmation of activity (either increased MIC or decreased zone diameter) ideally in a recombinant expression system, and ii) ≥2% difference in the amino acid sequence from another variant. We emphasize that TDase naming based on sequence similarity alone—even with the most stringent similarity thresholds—is likely to result in false positives and believe it is premature to name sequences as Tet(X) variants without functional validation. The importance of this kind of experimental confirmation of activity is highlighted by the fact that while our functional screening confirmed some sequences named based on similarity alone as active TDases (e.g., Tet(X20)), we also showed that single amino acid substitutions can result in a nonfunctional variants (Fig. 4c). For this reason, we did not include many sequences that have been reported as TDases without functional characterization in our MSA-based approach to avoid the risk of false positive sequences confounding our analyses.

To study the core sequence determinants of function we have restricted our analyses in the MSA-based approach to residues 100% conserved across every characterized TDase sequence. However, important insights could be gleaned using a less stringent cutoff, such as lower percent conservation, permitting some gaps, and including conserved substitutions. For example, several residues previously described as being involved in type 1 FAD or substrate binding were conserved in all but 1-3 sequences (G48, P308)^21^, and the entire GxGxxG FAD-binding motif would have been 100% conserved except for one sequence. Additional insights could be gleaned by analyzing type-specific conserved positions. For example, previously identified substrate-binding residues in Tet(X) and Tet(X7) (Q182, F214, and M365) were 100% conserved in all type 1 sequences but not identified in any type 2 sequence, suggesting a role in the different substrate specificities of the two enzyme types.

By identifying resides conserved across all known TDases we have set the foundation for future work focusing on the specific interactions and functional roles of each individual residue. This could provide valuable insights into the workings of this family of antibiotic enzymes and in turn how to block that functionality in order to rescue tetracycline antibiotic activity. Additionally, future work could include a more comprehensive, high-throughput mutagenic approach than alanine-scanning mutagenesis, such as deep mutational scanning which analyzes substitutions at all positions to all possible residues (DMS)^87^. This could shed light on type-specific conserved residues and evaluate residues which are conserved under slightly less strict criteria. These findings present both a broad survey of FMO sequences for TDase activity and a deep-dive into sequence-structure-function TDase enzymology, identifying new sequences and the functional requirements at specific positions.

## Methods

### Generating profile HMMs and sequence databases

Profile HMMs were generated using amino acid sequences of functionally characterized TDases (Supplementary Data 1). First, multiple sequence alignments of these input sequences were generated using Clustal Omega^88^, with *-t Protein* and *--outfmt=st*. Then profile HMMs were generated by inputting these MSAs into the *hmmbuild* function of HMMER^70^. Profiles were then run on protein sequence databases using *hmmsearch* function of HMMER^70^ with the *--tblout* and *-E 1e-10* flags.

The combined protein sequence database used in the first round (hmm01) was created by concatenating the NCBI Refseq Non-redundant Protein Database^52^, the Human Microbiome Reference Genome Database^53^, the CARD database^54^, and sequences from functional metagenomic selections^55–63^, totaling >321,000,000 amino acid sequences. To improve computational efficiency of analysis, this was divided into smaller fasta files of 1,000,000 sequences each to run *hmmsearch* in an array, with the results later merged. Second round HMMs were also run on this combined database, as well as a database of protein sequences from >115,000 metagenome-assembled genomes^64–69^, and the 460 sequences returned when NCBI BLAST was run with the Tet(50) amino acid sequence filtered to include just those with >35% amino acid identity to Tet(50) (http://blast.ncbi.nlm.nih.gov/).

The top 5000 hit sequences with the highest E-values were identified using the output *hmmsearch* results table. Their accession numbers of these hits were extracted from this table, de-duplicated to remove sequences with the same accession number due to overlapping sequence databases, and then the amino acid sequences of this sequence list was extracted from the input sequence databases. The resulting fasta files were then used to generate phylogenetic trees. Optimum HMM sequence score cut-off thresholds were determined using the *coords()* function of the pROC package^89^.

### Construction of phylogenetic trees and selection of HMM candidate sequences for screening

Phylogenetic trees were constructed using RAxML^90^ or fasttree^91^. For each, a multiple sequence alignment was created using Clustal Omega^88^ with *-t Protein* and *--outfmt=phy* for RAxML or *--outfmt=fa* for fasttree. Maximum-likelihood phylogeny was then constructed using RAxML with the following parameters: *raxmlHPC-PTHREADS -m PROTGAMMAJTT -f a -N 100 -p 12345 -x 54321*. Or using fasttree with *FastTreeMP* with the default parameters. Output *newick* trees were visualized and annotated using iTOL^92^.

In total we selected 50 candidate sequences for phenotypic testing. This was comprised of 26 candidate sequences in the first round (5 type 1, 3 type 2, 18 outside these clades), and 24 candidate sequences in the second round (type 1, 13 type 2, 10 outside) which met the following criteria:

- type 1: <99% identity to another type 1 sequence and <92% identity to another candidate sequence;
- type 2: <92% amino acid identity to another type 2 and another candidate sequence;
- outside: 40-80% amino acid identity to another type 1 or 2 sequence, and <92% amino acid identity to another candidate sequence. Sequences chosen were representative of the major clades formed by top-scoring hit sequences.

### Plasmids and strains

Candidate sequences of interest from the first HMM were synthesized and cloned into the *KpnI* and *BamHI* sites of the pZE21 plasmid (Expressys) by SynBio Technologies. Plasmid constructs were inserted into chemically competent *E. coli* MegaX (Expressys) by heat shock, prepared as 25% glycerol stocks, and stored at -80 °C. Genes in the pZE21 plasmid are constitutively expressed in the *E. coli* MegaX background. However, due to issues with escape mutants deleting pZE21’s promoter in the absence of tetracycline selection, we switched to using the *E. coli* DH5αZ1 + pZE24 plasmid system (Expressys). This plasmid’s P_lac/ara-1_ promoter is regulatable using IPTG and arabinose in the *E. coli* DH5αZ1 background^93^. All functionally confirmed sequences from the first HMM, candidate sequences of interest from the second HMM, as well as the Tet(X7) and Tet(50) sequences were cloned into the *KpnI* and *MluI* sites of the pZE24 plasmid. Assembly was performed using the HiFi DNA Assembly kit (NEB) as per the manufacturer’s instructions, with primers designed using NEBuilder. Plasmid constructs were inserted into chemically competent *E. coli* DH5αZ1 (Expressys) by heat shock, and prepared as 25% glycerol stocks. Cloning was verified by Sanger sequencing (GeneWiz). For expression and biochemical analyses, HMM hit sequences and TDase alanine-scanning mutants were cloned from pZE24 into the *NdeI* and *BamHI* sites of the pET28b(+) plasmid with a 6-His tag at the C-terminus. Assembly was performed using the HiFi DNA Assembly kit (NEB) as per the manufacturer’s instructions, with primers were designed using NEBuilder. Plasmid constructs were inserted into chemically competent *E. coli* BL21(DE3) by heat shock and prepared as 25% glycerol stocks. Cloning was verified by whole plasmid sequencing (Plasmidsaurus).

### Tetracycline screening and characterization with antibiotic susceptibility testing

Tetracycline screening by antibiotic susceptibility tests (AST) were performed with the microbroth dilution method, as per CLSI guidelines^94^. MIC panels were prepared in 96-well flat-bottom microplates (Corning) by two-fold serial dilution of tetracycline antibiotics in CAMH + 50 μg/mL kanamycin broth, then stored at -80 °C for <6 months. On the day of the experiment, MIC panels were thawed at room temperature, overnight cultures were grown to exponential phase (OD_600_ = 0.3–0.8, ~2.5 h) in fresh media containing 1 mM IPTG, then diluted to a standard concentration and inoculated into each panel at a 1:1 ratio. Each well had a final concentration of 50 μg/mL kanamycin, 1 mM IPTG, ~5 × 10^5^ CFU/mL cells (0.5 MacFarland), and variable concentrations of the antibiotic of interest. (Screening done with pZE21 strains did not contain IPTG.) Each test was performed in triplicate, with no-antibiotic and no-cell control wells. For high expression from the *P*_*lac/ara-1*_ promoter, 1 mM IPTG fully relieves repression by LacI^93^. Inoculated panels were incubated at 37 °C for 20 h with shaking, then scored by eye and using a plate reader.

### Gene naming scheme

In line with previously established criteria for classifying AR genes broadly^25,86^, we have named our sequences using the following parameters: (i) must have experimental confirmation of activity, (ii) new *tet* genes must have <79% amino acid identity with all previously characterized *tet* gene amino acid sequences, (iii) sequences with 79-98% amino acid identity to a previously characterized *tet* gene are designated as a gene variant, and (iv) sequences with 98-99.9% amino acid identity (i.e. at least one substitution) to a previously characterized *tet* variant are designated as a gene sub-variant. Gene variants are indicated with a number, either directly following X for type 1 s (e.g., Tet(X7)) or following a dash for type 2 s (e.g., Tet(56-2)). Gene subvariants are indicated with the number followed by a decimal point (e.g., Tet(X7.2)). We have additionally compared our functional sequences to both characterized Tet(X) variants, and variant sequences which have been named in previous reports based on sequence similarity but without functional confirmation of activity.

### Purification of TDases

Cultures of *E. coli* BL21(DE3) + pET28b(+) strains containing HMM hit sequences and TDase alanine-scanning mutants were prepared at 37 °C in lysogeny broth containing kanamycin (Kan) at 0.05 mg/mL (final concentration); once the culture reached an OD_600_ of ~0.6, the cells were cooled to 4 °C. Protein expression was induced by the addition of 0.5 mM IPTG (final concentration), and cells were grown at 15 °C for 18 h. To harvest protein, the induced cells were pelleted by centrifugation at 5000 RPM for 15 min (4 °C) and resuspended in 40 mL of cold lysis buffer (50 mM K_2_HPO_4_, 500 mM NaCl, 20 mM imidazole, 10% glycerol, 5 mM BME, pH 8.0) containing SIGMAFAST© protease inhibitor (Millipore-Sigma). Cell suspensions were flash frozen in liquid nitrogen and stored at −80 °C or immediately lysed. Frozen cell suspensions were thawed and lysed by sonication, and the resultant lysate was clarified via ultracentrifugation at 30,000 rpm for 35 min at 4 °C. The clarified supernatant was transferred to a fritted column containing washed and equilibrated Ni-NTA resin and incubated for 30–60 min with gentle rocking at 4 °C. The resin was then washed with lysis buffer (2 × 40 mL), and the protein was eluted from the resin with elution buffer (5 × 10 mL elutions, 50 mM K_2_HPO_4_, 500 mM NaCl, 5 mM BME, 300 mM imidazole, 10% glycerol, pH 8.0). Fractions containing the desired proteins (as judged by SDS-PAGE analysis) were combined and transferred to a 10,000 molecular weight cutoff (MWCO) Snakeskin dialysis tubing (ThermoScientific) and equilibrated in dialysis buffer (50 mM K_2_HPO_4_ pH 8.0, 150 mM NaCl, 1 mM DTT) overnight. Dialyzed protein solutions were concentrated using a 30,000 MWCO Amicon centrifugal filter (Millipore-Sigma), and the concentrated protein solution was flash frozen as beads in liquid nitrogen (50 μL portions) and stored at −80 °C.

### Apparent steady-state kinetics of substrate inactivation

Reactions were prepared in 100 mM TAPS buffer at pH 8.5 with varying amounts of substrate (typically 0-97 μM), 504 μM NADPH, 5.04 mM MgCl2, and 0.4 μM TDase. Reactions were initiated by the addition of TDase and were monitored continuously via optical absorbance spectroscopy at 400 nm for 2 min (performed in duplicate or triplicate as independent trials). Initial enzyme velocities were determined by linear regression using Agilent Cary WinUV Software over the linear range of the reaction (typically between 0 and 1 min), plotted against the concentration of the substrate, and fitted to the Michaelis−Menten equation using GraphPad Prism 6.

### Crystallization, data collection, and structure refinement

Tet(56-2) was concentrated to 20 mg/mL in 10 mM HEPES, pH 7.4, 100 mM NaCl and 5 mM DTT and crystallized by hanging drop vapor diffusion at 18 °C in 0.15 M magnesium formate and PEG 15%. Crystals were transferred to the reservoir solution containing 20% glycerol for 15–30 s and flash-cooled in the liquid nitrogen. Tet(56-3) was concentrated to 20 mg/mL in 10 mM HEPES, pH 7.4, 100 mM NaCl and 5 mM DTT, and crystallized by hanging drop vapor diffusion at 18 °C in 0.1 M MES pH 6.0 and 5%(w/v) PEG 3000. Crystals were transferred to the reservoir solution containing 30%(v/v) PEG 200 and flash-cooled in liquid nitrogen. Diffraction data were collected at 100 K at the GM/CA 23IDD beamline, Advanced Photon Source (APS), Argonne National Laboratory. All data processing and structure analysis were performed using SBGrid. Diffraction data were reduced and scaled using XDS^95^. Tet(56-2) and Tet(56-3) structures were solved by molecular replacement using PHASER^96^ with the substrate-free Tet(56) structure PDB 5TUE^23^ as search models. Structure refinement and model building were performed in PHENIX^97^ and Coot^98^. The final model was validated using the MolProbity server^99^. The loop regions encompassing residues 142–146 and 356–360 showed reasonable backbone density but poor sidechain density resulting in RSR Z-score of 4.4% and 8.7% for these residues.

### Determination of apparent anhydrotetracycline IC_50_ values

All experiments were prepared open to air in non-degassed buffer solutions at room temperature. Half-maximal inhibitory concentrations (IC_50_) for the inhibition of Tet(56-2) and Tet(56-3) were determined from the velocities of substrate degradation in the presence of varying concentrations of inhibitor. Reaction samples were prepared in 100 mM TAPS buffer (pH 8.5) with 504 μM NADPH, 5.04 mM MgCl_2_, 25.3 μM substrate, varying concentrations of inhibitor (0-227 μΜ), and 0.4 μM enzyme (final working concentrations). Reactions were initiated by the addition of enzyme and were monitored continuously via optical absorbance spectroscopy at 400 nm for 2 min (performed in triplicate as independent trials). Initial enzyme velocities were determined by linear regression using Agilent Cary WinUV Software over the linear range of the reaction (0 to 1 min). The velocities were plotted against the logarithm of inhibitor concentration, and apparent IC_50_ values were determined using nonlinear regression analysis in GraphPad Prism 6. Each set of experiments included a no-TDase control reaction which was used as the full enzyme inhibition velocity and assigned to inhibitor concentration of 1 × 10^15^, and a no-inhibitor control which was assigned an inhibitor concentration of 1 × 10^–15^. A no-tetracycline control was also performed to search for potentially competitive background signals generated from the enzymatic degradation of the inhibitor. For all inhibitor-enzyme combinations, the initial velocities of the no-tetracycline controls were negligible.

### Checkerboard microbroth dilution inhibition assays

For checker-board whole cell inhibition assays, anhydrotetracycline was two-fold serially diluted in a constant concentration of tetracycline. Liquid cultures of each strain were grown to exponential phase then diluted to a standard concentration (OD_600_ = 0.0015, which is equivalent to double ~5 × 10^5^ CFU/mL) and inoculated into each panel at a 1:1 ratio. Thus, each well had a final concentration of 50 μg/mL kanamycin, 1 mM IPTG, ~5 × 10^5^ CFU/mL (0.5 MacFarland) cells, and variable concentrations of the antibiotic of interest or anhydrotetracycline. Each strain-antibiotic/inhibitor combination was tested in triplicate, along with no-drug and no-cell controls. Inoculated panels were sealed with Breathe-Easy membranes (Sigma-Aldrich) and incubated at 37 °C for 20 h. Panels were scored by absorbance measurements at 600 nm (OD600) using the Synergy H1 microplate reader (Biotek Instruments, Inc).

### Identification of conserved positions in amino acid sequence alignment

We collected 114 TDase sequences whose activity as tetracycline resistance genes had been confirmed by isolate screening, functional metagenomics, and/or recombinant expression. Conserved amino acid positions were identified using ConPosER (https://github.com/kevinsblake/ConPosER) with *msa* = *”ClustalOmega”*. The *composer_id()* function calls the *msa* R package^100^, and by default ignores columns with >30% gaps as these are unlikely to be functionally important. Geneplots were generated with the *conposer_plot()* function with *gap.lim* = *0.05*, ignoring columns with >5% gaps. Positions were mapped onto the 3D crystal structures of Tet(X7) and Tet(50) in PyMOL^101^. For the broader FMO sequence analysis, we also included 14 class A FMO sequences with solved crystal structures. These were, PDB: 1FOH, 2DKH, 2R0P, 3IHG, 2QS1, 2BRY, 1PBE, 4A6N, 4K22, 4J31, 2VOU, 3RP6, 4BJZ, and 2RGJ.

### Alanine-scanning mutagenesis

Alanine scanning mutagenesis was performed using the QuikChange® Lightning Site-Directed Mutagenesis Kit (Agilent) as per the manufacturer’s instructions. Mutagenic primers were designed using the Quikchange® Primer Design Program to mutate the codon of the conserved position to GCC alanine (Supplementary Data 1). Template pZE24 + *tet*(X7) and pZE24 + *tet*(50) was purified using the QIAprep® Spin Miniprep Kit (Qiagen). PCR was performed on template Tet(X7) and Tet(50) in pZE24 with an annealing temp of 60 °C and an extension time of 1 min 48 s. PCR products were *DpnI* digested as per the manufacturer’s protocol, then inserted into chemically competent *E. coli* DH5αZ1 (Expressys) by heat shock, and prepared as 25% glycerol freezer stocks. Successful mutagenesis was verified using Sanger sequencing (GENEWIZ).

### Thermal stability assays

The stability of wild-type and mutant proteins was assessed using the QuantStudio™ 3 Real-Time PCR System and the Protein Thermal Shift™ Starter Kit (Applied Biosystems), as per manufacturers’ instructions. The fluorescence of the solution was then measured over a range of temperatures (23–99 °C), and the resultant data were analyzed using the Protein Thermal Shift™ Software v1.4 (Applied Biosystems).

### Statistics and reproducibility

AST and checkerboard inhibition assays were performed in triplicate. X-ray structural analysis statistics including the number of crystals used per data set are included in Supplementary Data 1. Standard statistical analysis for X-ray diffraction data processing and analysis were adhered to and presented in Supplementary Data 1. Enzyme assays were performed in two or three independent trials.

### Supplementary information

Supplementary Information

Description of additional supplementary files

Supplementary Data 1

Supplementary Data 2

## Data Availability

Source data underlying the graphs and charts are provided in Supplementary Data 2. X-ray structures available on PDB under PDB IDs 8TWG and 8TWF.

## Code availability

Analysis scripts are available at: https://github.com/dantaslab/2023_Blake_TDase-HMM-mut and deposited at 10.5281/zenodo.10636944^102^.
