# Supplementary material for: Sequence-structure-function characterization of the emerging tetracycline destructase family of antibiotic resistance enzymes
Source: Commun Biol. 2024 Mar 16;7:336. doi: 10.1038/s42003-024-06023-w (PMC10944477; doi:10.1038/s42003-024-06023-w)
Supplement: Supplementary file 1 — Supplementary Information [file 42003_2024_6023_MOESM1_ESM.pdf]

Supplementary figures and figure legends

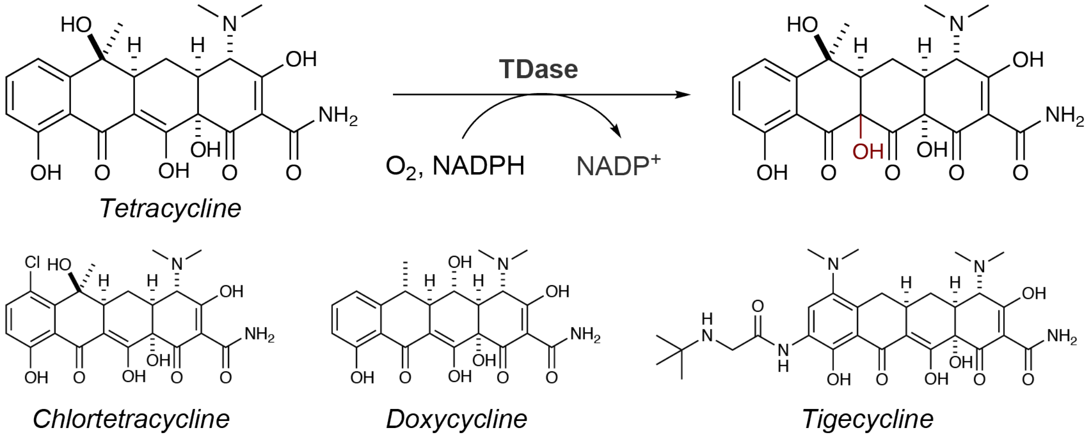

Supplementary Figure 1. TDase catalytic reaction, and chemical structures of antibiotics used.

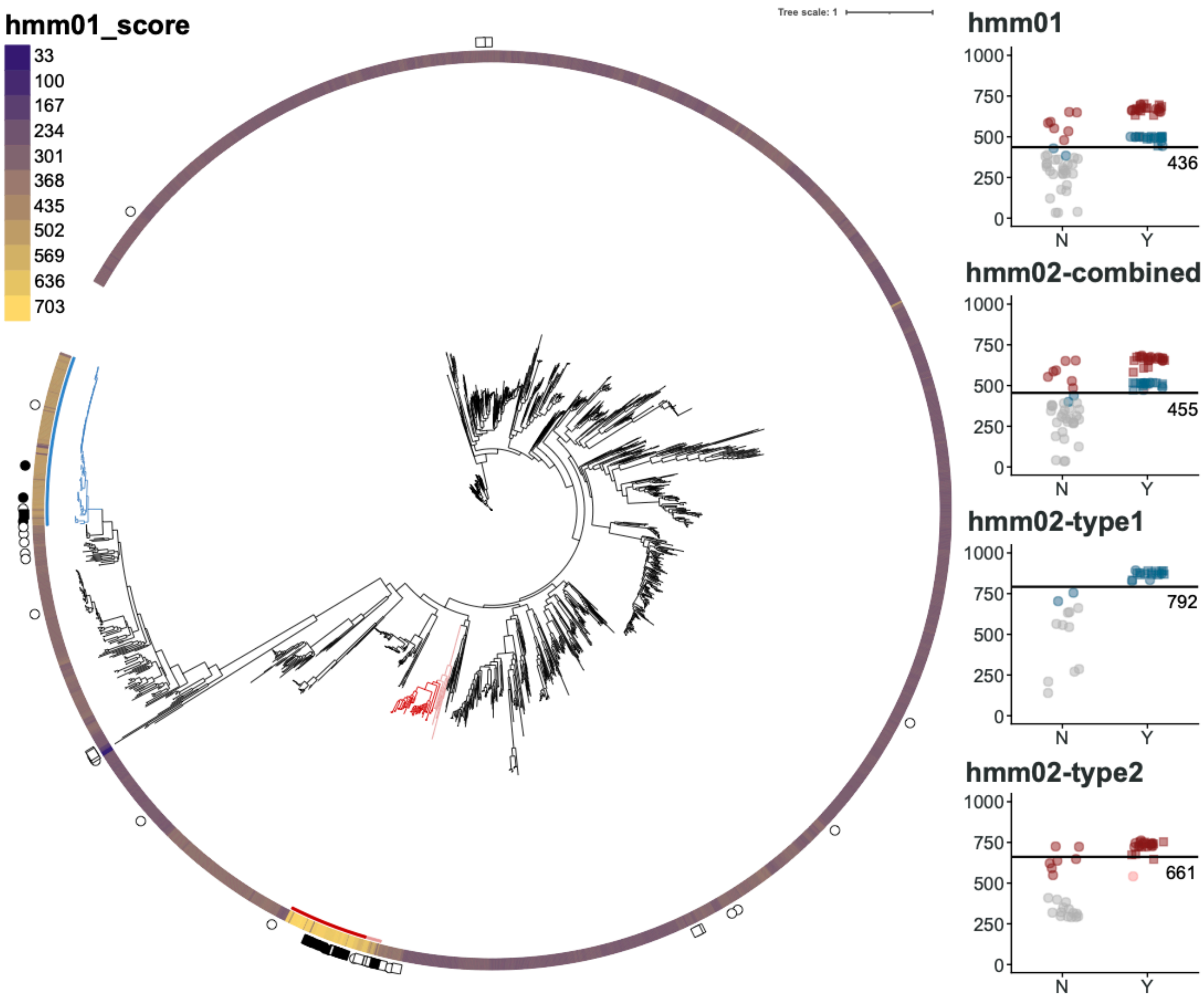

Supplementary Figure 2. (Left) Phylogenetic tree comprised of characterized TDase sequences, all synthesized HMM hit sequences, and the top 2000 sequences with the highest E-values from hmm01 run on the combined sequence database. Generated with fasttree. Color bar ring represents the sequence score run against hmm01. Branch color and colored range indicates TDase clade (type 1 = blue; type 2 = red; expanded type 2 = pink). Circles indicate tested hmm01-predicted sequences: filled circle = functional (i.e. confers resistance to tetracycline); empty circle = does not confer resistance. Squares indicate tested hmm02-predicted sequences: fille square = functional; empty square = does not confer resistance. **(Right)** Dotplot of the sequence bit score of synthesized HMM hit sequences (circle) and characterized TDase sequences (square) for different profile HMMs. Points colors indicate TDase clade as defined prior to HMM search (type 1 = blue; type 2 = red; expanded type 2 = pink; neither = gray), binned by if they're nonfunctional (N) or functional (Y). For the type-specific plots, only HMM hit sequences with >40% amino acid

identity to sequences in the given type are shown. Horizontal line represents the optimal threshold for using sequence bit score to determine whether an HMM hit sequences is functional (hmm02-type1 specificity and sensitivity = 1; hmm02-type2 specificity = 0.91, sensitivity = 0.91).

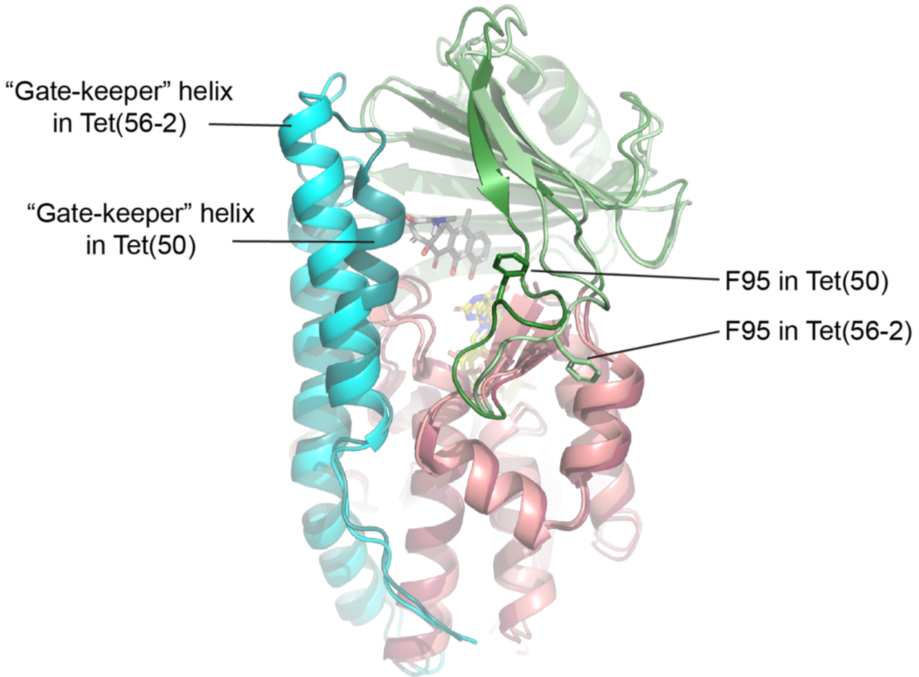

Supplementary Figure 3. Structural comparison of Tet(56-2) with Tet(50) (PDB ID: 5TUF). Tet(50) chain B (PDB: 5TUF) was superposed to Tet(56-2) X-ray structure. FAD-binding domain is shown in salmon and raspberry, substrate-binding domain is shown in pale green and forest, C-terminal bridge helix is shown in cyan and teal for Tet(56-2) and Tet(50), respectively. Bound FAD (yellow) and anhydrotetracycline (gray) molecules are shown in ball-and-stick.

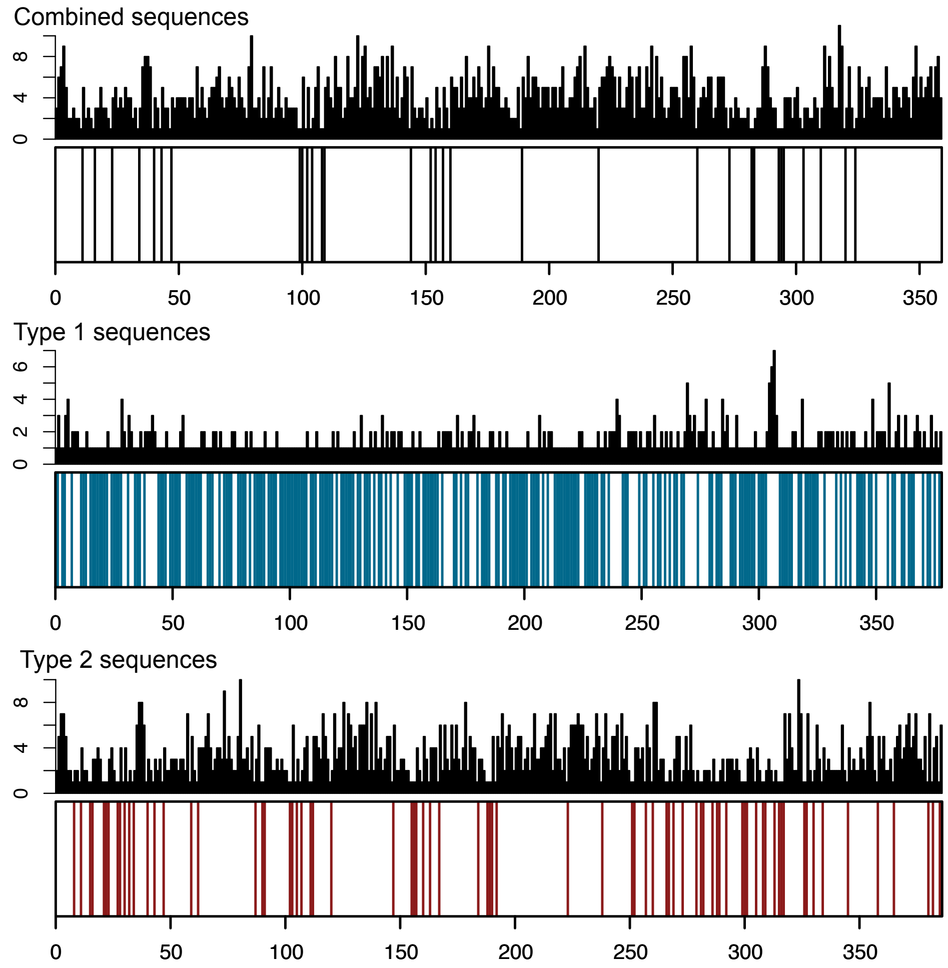

Supplementary Figure 4. Geneplots of the MSAs comprised of **(top)** all 114 TDase sequences, **(middle)** just the 91 type 1 sequences, and **(bottom)** just the 23 type 2 sequences. Colored lines (black, blue, red) represent positions 100% conserved in the MSA. The combined MSA had 31 conserved positions, type 1 had 238 conserved positions, and type 2 had 76 conserved positions. Geneplot MSAs have been filtered to mask positions with a gap in >10% of sequences in the MSA. Barplots above each geneplot denotes the number of amino acids observed at each position for each MSA, with a value of 1 corresponding to 100% conserved positions, and also indicated with a line in the geneplot.

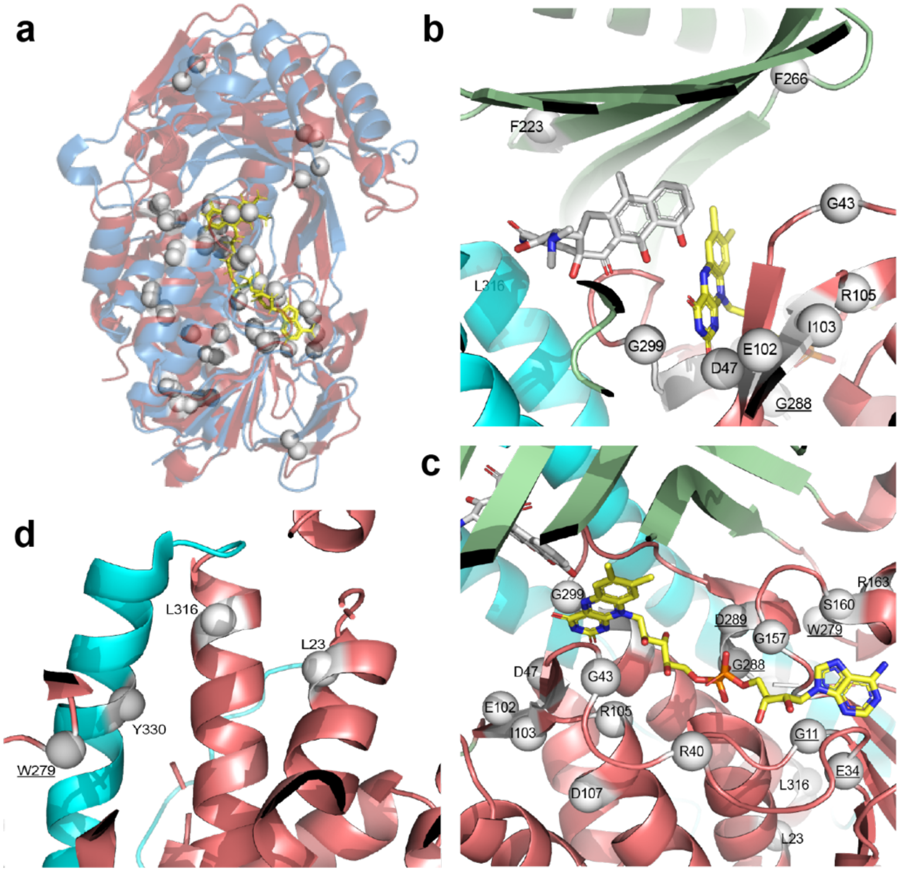

Supplementary Figure 5. (a) Overlay of the Tet(X7) monomer A (PDB 6WG9; blue) and Tet(50) monomer A (PDB 5TUE; red) crystal structures with the 31 conserved positions shown as white main chain spheres. FAD shown in yellow stick. **(b)** Structure of anhydrotetracycline binding pocket, **(c)** FAD-binding pocket and **(d)** hydrophobic core of Tet(50) monomer B (PDB: 5TUF). The anhydrotetracycline shown in gray stick. The labelled residues are 100% conserved in MSA, and the underlined residues are true essential (alanine mutants lost activity to all drugs in both enzymes).

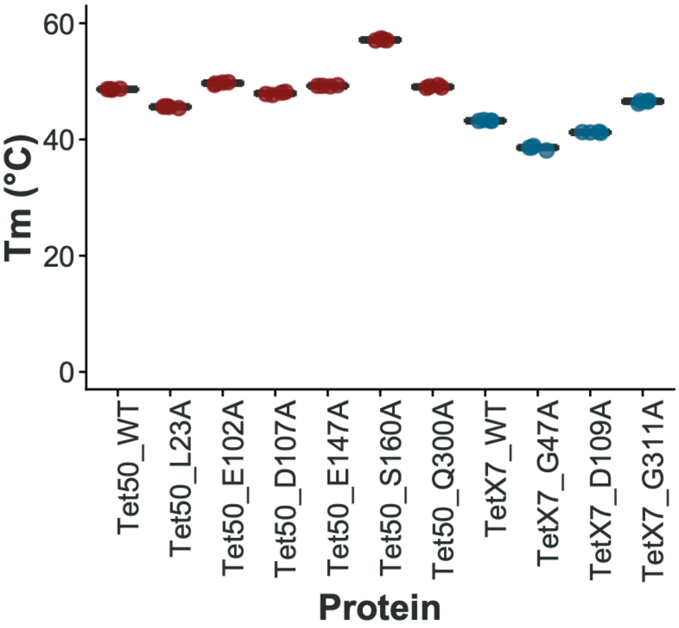

Supplementary Figure 6. Thermal stability of WT Tet(X7) and Tet(50) along with mutants at conserved positions. Points represent values from four technical replicates, colored by TDase type the protein belongs to (blue = type 1, red = type 2). Black boxplots behind points show the mean of four replicates, with upper and lower hinges corresponding to the first and third quartiles.
