# Supplementary material for: Sequence-structure-function characterization of the emerging tetracycline destructase family of antibiotic resistance enzymes
Source: Commun Biol. 2024 Mar 16;7:336. doi: 10.1038/s42003-024-06023-w (PMC10944477; doi:10.1038/s42003-024-06023-w)
Supplement: Supplementary file 2 — Description of additional supplementary files [file 42003_2024_6023_MOESM2_ESM.pdf]

Description of Additional Supplementary Files

File name: Supplementary data 1

Description: Supplementary Tables

File name: Supplementary data 2

Description: Source data underlying graphs and charts presented in the main figures
